# Isolation and characterization of a new cold-active protease from psychrotrophic bacteria of Western Himalayan glacial soil

**DOI:** 10.1038/s41598-021-92197-w

**Published:** 2021-06-17

**Authors:** Saleem Farooq, Ruqeya Nazir, Shabir Ahmad Ganai, Bashir Ahmad Ganai

**Affiliations:** 1 Department of Environmental Science, University of Kashmir, Srinagar, Jammu and Kashmir 190006 India; 2 Microbiology Research Laboratory, Centre of Research for Development (CORD), University of Kashmir, Hazratbal, Srinagar, India Jammu and Kashmir 190006; 3 Division of Basic Sciences and Humanities, FoA, SKUAST-Kashmir, Srinagar, Jammu and Kashmir 193201 India

**Keywords:** Biotechnology, Computational biology and bioinformatics, Microbiology

## Abstract

As an approach to the exploration of cold-active enzymes, in this study, we isolated a cold-active protease produced by psychrotrophic bacteria from glacial soils of Thajwas Glacier, Himalayas. The isolated strain BO1, identified as *Bacillus pumilus*, grew well within a temperature range of 4–30 °C. After its qualitative and quantitative screening, the cold-active protease (Apr-BO1) was purified. The Apr-BO1 had a molecular mass of 38 kDa and showed maximum (37.02 U/mg) specific activity at 20 °C, with casein as substrate. It was stable and active between the temperature range of 5–35 °C and pH 6.0–12.0, with an optimum temperature of 20 °C at pH 9.0. The Apr-BO1 had low K_m_ value of 1.0 mg/ml and V_*max*_ 10.0 µmol/ml/min. Moreover, it displayed better tolerance to organic solvents, surfactants, metal ions and reducing agents than most alkaline proteases. The results exhibited that it effectively removed the stains even in a cold wash and could be considered a decent detergent additive. Furthermore, through protein modelling, the structure of this protease was generated from template, subtilisin E of *Bacillus subtilis* (PDB ID: 3WHI), and different methods checked its quality. For the first time, this study reported the protein sequence for psychrotrophic Apr-BO1 and brought forth its novelty among other cold-active proteases.

## Introduction

Psychrophilic and psychrotrophic bacteria inhabit almost all permanently cold habitats, from polar to non-polar glaciers and deep sea^1,2^. The prime significance of these bacteria stems from their ability to survive and retain functionality in cold temperatures and grow optimally even at warm temperatures^3^. These bacteria offer an immense natural resource of cold-active enzymes that function at low temperatures (10–20 °C). Indeed, these bacteria have been targeted for their industrial and biotechnological potential, for they provide energy-saving and economically beneficial alternatives to the enzymes from mesophilic homologues at low temperature^4,5^. These enzymes have evolved with increased conformational flexibility stemming from weakened non-covalent interactions, like salt bridges, hydrophobic interactions, aromatic-aromatic interactions and hydrogen bonding^6^. Among various enzymes, alkaline proteases that are usually active at different pH and temperature ranges^7,8^ are ranked high in commercial applications. Capable of producing large amounts of extracellular serine proteases, *Bacilli* are the chief source of almost all major serine alkaline proteases used in detergents^9^. While the contribution of bacterial alkaline proteases to total protease market share stands at 60%^10–12^, the data related to cold-active alkaline proteases from psychrotrophs is essentially missing^4^.

Nonetheless, a handful of previous studies have successfully isolated and characterized the small number of alkaline cold-active proteases from *Bacillus* sp. and demonstrated their application in detergent industries^4,13,14^. Though numerous cold-active proteases have been isolated from different cold environments, only a few are alkaline and appropriate as detergent additives^15^. In addition, crystal structures of numerous alkaline serine proteases from *Bacilli* have been identified^16–20^. The commercially important detergent proteases like subtilisin BPNʹ^18^, Carlsberg^21^, Savinase and Esperase^19,22^, are stable at higher pH (≥ 9) and temperature (25–40 °C), but the majority of the other proteases are comparatively unstable. Notably, in bleach-based detergents, these proteases do not withstand the oxidizing agents like hydrogen peroxide (H_2_O_2_), surfactants like Tween-80 and sodium dodecyl sulfate (SDS)^23^.

Despite advancement, the dearth of novel alkaline cold-active proteases characterized by being active at low temperatures, stable in the presence of metal ions, surfactants, organic solvents and oxidizing agents still exists. The main aim of the present study was to purify and characterize serine alkaline protease from a psychrotrophic bacterium that can catalyze reactions at low temperature and be used as an industrial additive. The cultural conditions like media, temperature, pH, and incubation time were modified to match psychrotrophic bacterium and obtain maximum yield at conditions suitable for the strain. This study successfully characterized and purified cold-active serine alkaline protease from psychrotrophic *Bacillus pumilus* BO1 isolated from western Himalayan glacial soil.

## Results

### Bacterial isolation and screening

The strain BO1 was selected among other isolated strains for further study after it displayed the highest proteolytic activity during primary screening. The isolated BO1 strain was able to grow at temperatures between 4 and 30 °C with the optimum growth at 20 °C.

### Identification and qualitative screening of BO1 strain

**Figure 1:**
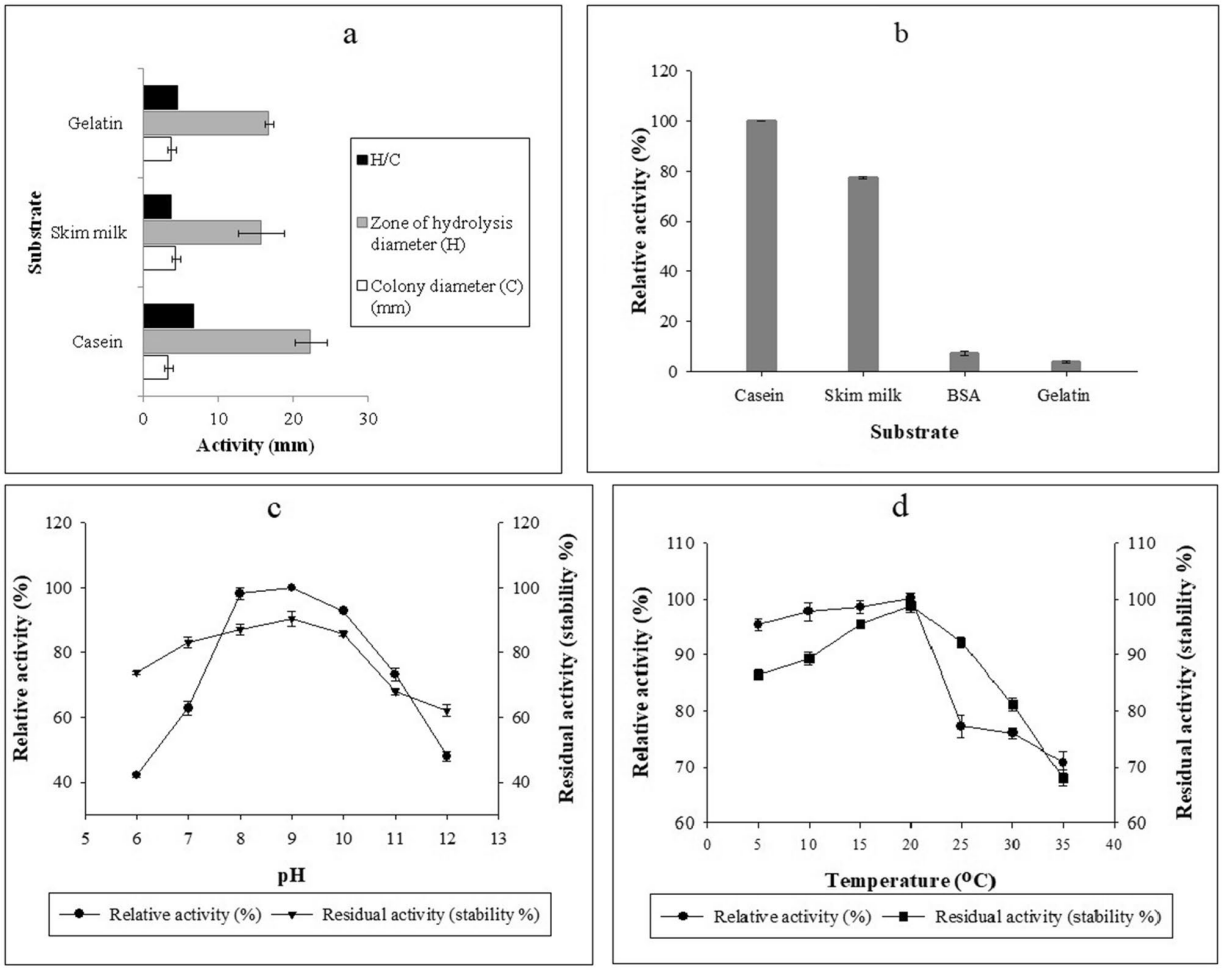
(**a**) Qualitative screening of BO1 strain for its proteolytic activity on three different substrates. (**b**) Substrate efficacy of purified Apr-BO1. (**c**) Effect of pH on the activity and stability of purified cold-active Apr-BO1. (**d**) Effect of temperature on the activity and stability of purified Apr-BO1.

The colony morphology and physiological characteristics of BO1 strain were round with undulate margin, flat elevation, and spore-forming rod-shaped Gram-positive aerobe. The amplified 16S rRNA gene sequence showed a 100–98% sequence identity to *Bacillus pumilus* when blasted against the nucleotide sequence database at NCBI. The 16S rRNA gene sequence of BO1 strain was submitted in NCBI GenBank under Accession number MN094861. The 16S rRNA phylogenetic tree (Supplementary Fig. S1) of psychrotrophic *Bacillus pumilus* BO1 showed significant similarity with other highly-homologous *Bacillus pumilus* species by sharing the same branch in the phylogenetic tree. The BO1 strain showed highest protease activity (zone of hydrolysis) on casein (Supplementary Fig. S2) out of three different media used (Fig. 1a).

### Purification and molecular mass determination of cold-active protease

**Figure 2:**
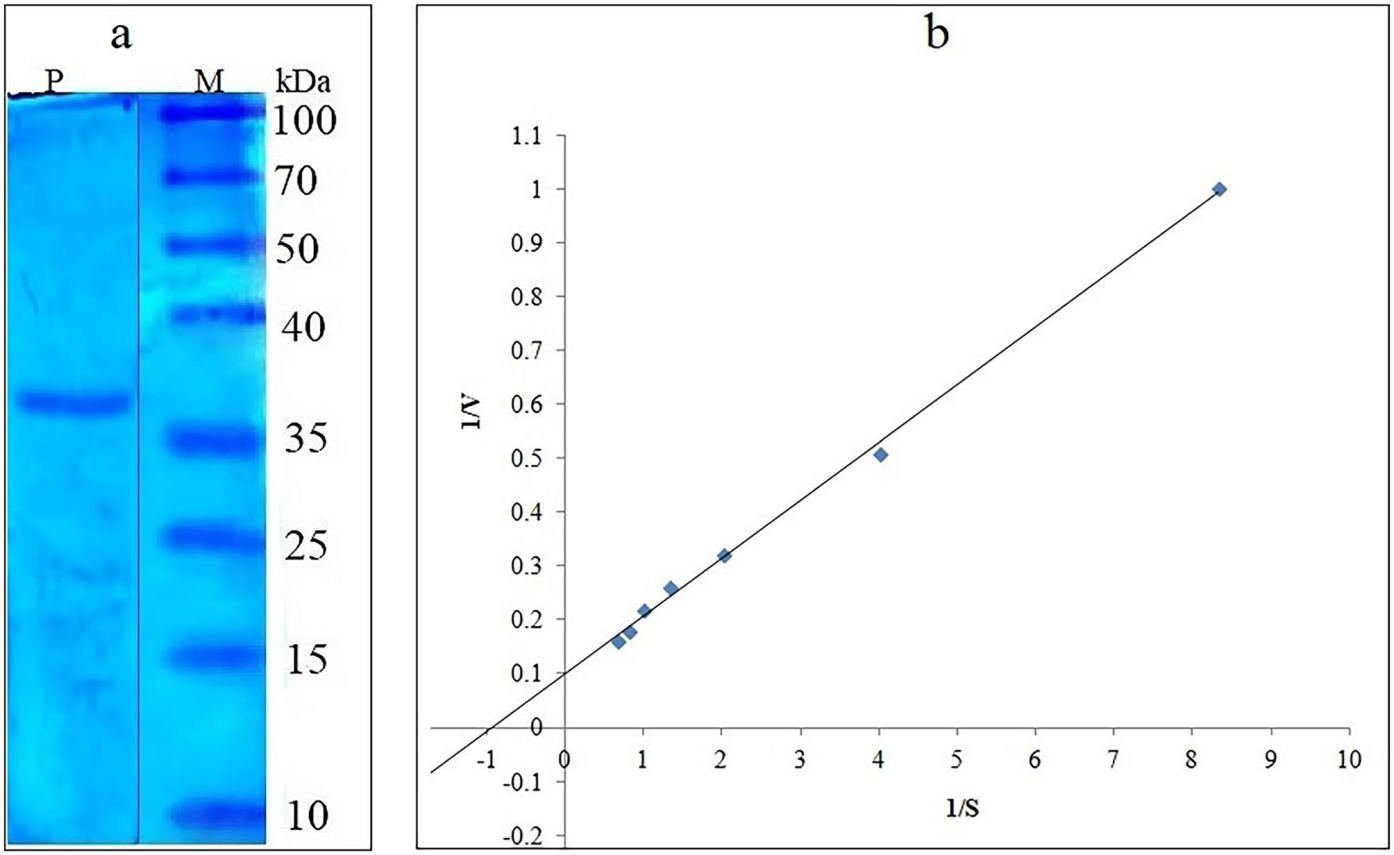
(**a**) SDS-PAGE of purified Apr-BO1 (lane P). Lane M, molecular protein marker. (**b**) Lineweaver–Burk plot of purified cold-active Apr-BO1. The kinetic parameters (K_m_ and V_max_) were studied by varying casein concentration from 0.02 to 0.22 g%. The original image (**a**) was cropped; the full-length image is presented as Supplementary Fig. S6b along with SDS-PAGE analysis of crude extracts (Supplementary Fig. S6a).

The protease (Apr*-*BO1) achieved a 4.43-fold increase in its purification with 80% ammonium sulphate saturation, followed by dialysis, which increased the purification by 17.63-folds. 92.55-fold purification was achieved with Sephadex G-50 superfine (Sigma, USA) gel filtration column. The purification profile of cold-active protease is given in Table 1. Further, the single-band appearance on SDS-PAGE indicated that purified Apr-BO1 had a molecular mass of 38 kDa (Fig. 2a).

**Table 1:** Purification steps of Apr-BO1.

Purification	Total activity (U)	Total protein (mg)	Specific activity (U/mg)	Purification folds
Culture filtrate	92.4 ± 7.6	229.3 ± 26.73	0.40	1
Ammonium sulphate precipitation	316.80 ± 19.28	178.6 ± 14.05	1.77	4.43
Dialyzed fraction	789.12 ± 18.19	112.0 ± 2.68	7.05	17.63
Sephadex G-50	1085.76 ± 34.5	29.33 ± 0.61	37.02	92.55

The values are the mean of three replicates ± standard deviation.

### Effect of pH and temperature on activity and stability of the purified enzyme

The enzyme showed an increase in its activity from pH 6.0, reaching its optimum activity (100%) at pH 9.0, after which the activity started declining with an increase in pH up to 12.0 (Fig. 1c). Compared to protease activity at pH 9.0, it showed 42.13, 62.83 and 98.20% activity at pH 6.0, 7.0, and 8.0, respectively. Further, when the pH of the reaction mixture was increased towards alkalinity, Apr-BO1 displayed the relative activity of 92.8, 73.23 and 48.03% at pH 10.0, 11.0, and 12.0, respectively. As a result, Apr-BO1 was classified as an alkaline protease. On the other hand, the pH-dependent enzyme stability revealed that Apr-BO1 was stable over the pH range 6.0–12.0 (Fig. 1c), with only a 7.33–3.35% reduction in its activity at pH 7.0 and 8.0, respectively (concerning activity at pH 9.0). When incubated at pH 12.0, however, there was a 28.0% reduction in its activity.

The temperature-dependent enzyme activity showed an increase in relative activity with an increase in temperature from 5 to 20 °C, with optimum activity being observed at 20 °C. Further, increase in incubation temperature above 20 °C sharply decreased the enzyme activity, with approximately 71.0% activity being retained at 35 °C concerning 100% relative activity at 20 °C. While as the enzyme thermostability varied from 86.0 to 99.0% at 5–20 °C, respectively, which reveals that Apr-BO1 is stable at low temperatures. The enzyme showed decreased stability above 20 °C and retained average stability of 91.0, 80.0 and 67.0% (with respect to optimum stability at 20 °C) at 25 °C, 30 °C and 35 °C, respectively (Fig. 1d).

### Effect of metal ions, surfactants, organic solvents, inhibitors and oxidizing agents on the activity of purified cold-active protease

The results of the metal ion effect on the activity of protease are given in Table 2. As it is evident, Fe^2+^ showed an increase in the relative activity of the enzyme by 13.0% concerning control, which was better than Cu^2+^ (9%) at a lower concentration (1.0 mM). Further, Cu^2+^ and Zn^2+^ also increased the enzyme activity up to 5.0 mM while as the activity decreased at 10.0 mM concentration of Cu^2+^ and Zn^2+^. However, Fe^2+^ did not show any significant effect on enzyme activity at higher concentration. Mn^2+^ showed a gradual increase with increasing concentration and exhibited a 16.02% increase in enzyme activity at 10.0 mM concentration. Ca^2+^ also increased the enzyme activity by 12.5, 19.0 and 23% at 1.0 mM, 5.0 mM and 10.0 mM, respectively. On the other hand, metal ions like Hg^2+^, Co^2+^ and Cd^2+^ decreased the enzyme activity with increasing concentration (Table 2). Besides, the protease showed enough activity in the presence of surfactants, oxidizing agents (Table 3) excluding, SDS which decreased the enzyme activity by 16.74%, 28.6% at 0.5% and 1.0% concentrations, respectively. Cold-active protease also showed good tolerance towards the oxidizing agent (H_2_O_2_). The inhibitor EDTA showed a 32.0% decrease at 1.0 mM concentration, while a sharp decline of 98.0% in enzyme activity was observed at 10.0 mM EDTA concentration. The organic solvents, xylene (100%), did not affect the enzyme activity, while toluene (95.0%) and benzene (86.0%) had a moderate effect on it. In addition, the enzyme showed good stability with all the organic solvents tested (Table 4).

**Table 2:** Effect of metal ions and inhibitor on the activity of purified cold-active Apr-BO1 at three different concentrations.

Metal ions	1.0 mM (%)	5.0 mM (%)	10.0 mM (%)
Control	100 ± 0.0	100 ± 0.0	100 ± 0.0
Mn^2+^	103.50 ± 2.2	114.00 ± 0.3	115.94 ± 0.1
Mg^2+^	100 ± 1.0	112.3 ± 0.92	100.67 ± 2.1
Hg^2+^	59.58 ± 0.6	16.03 ± 0.6	3.90 ± 0.5
Cd^2+^	63.07 ± 0.9	60.71 ± 0.5	30.40 ± 1.0
Co^2+^	81.26 ± 1.1	23.34 ± 2.9	5.37 ± 0.7
Fe^2+^	113.37 ± 0.8	100.35 ± 0.7	99.71 ± 0.6
Cu^2+^	108.92 ± 1.1	106.22 ± 1.4	96.03 ± 0.8
Zn^2+^	106.42 ± 0.8	105.09 ± 0.9	51.58 ± 3.6
Ca^2+^	112.53 ± 1.0	119.04 ± 2.2	123.69 ± 1.6
EDTA	77.7 ± 0.7	35.17 ± 0.7	11.5 ± 1.3

The values are the mean of three replicates ± standard deviation.

**Table 3:** Effect of different surfactants and oxidizing agent on the activity of purified cold-active Apr-BO1.

Surfactants	Relative activity (%)	
	0.5%	1.0%
Control	100 ± 0.00	100 ± 0.00
Tween 80	60.06 ± 1.1	63.70 ± 5.3
Triton X-100	35.4 ± 1.2	0.00 ± 0.0
SDS	83.26 ± 10.8	71.4 ± 1.2
H_2_O_2_	103.5 ± 3.5	105.8 ± 4.3

**Table 4:** Effect of organic solvents on the activity and stability of purified cold-active Apr-BO1.

Organic solvents	Residual activity (stability %)	Relative activity (%)
Control	100 ± 0.00	100 ± 0.00
Benzene	79.53 ± 0.50	86.97 ± 0.55
Toluene	96.47 ± 0.47	95.1 ± 0.36
Xylene	92.97 ± 0.35	100.73 ± 1.27

The values are the mean of three replicates ± standard deviation.

### Substrate efficacy

The protease, Apr-BO1, showed relative activity towards casein, skim milk, bovine serum albumin (BSA) and gelatin to a diverse extent. When standardized to casein (100%), the activity of enzyme towards these substrates was 77.91% for skim milk, 6.4% for BSA, and 3.34% for gelatin (Fig. 1b).

### Enzyme kinetics (K_m_ and V_max_) of purified Apr-BO1 towards casein

The rate of reaction exhibited a sharp increase with the increase in casein concentration up to 0.15 g%, after which the reaction rate stabilized. Apr-BO1 displayed K_m_ of 1.0 mg/ml (4.16 × 10^–5^ M) and V_max_ of 10.0 µmol/ml/min against casein, showing its high affinity and efficient catalytic role (Fig. 2b).

### Protease gene identification and sequence analysis

**Figure 3:**
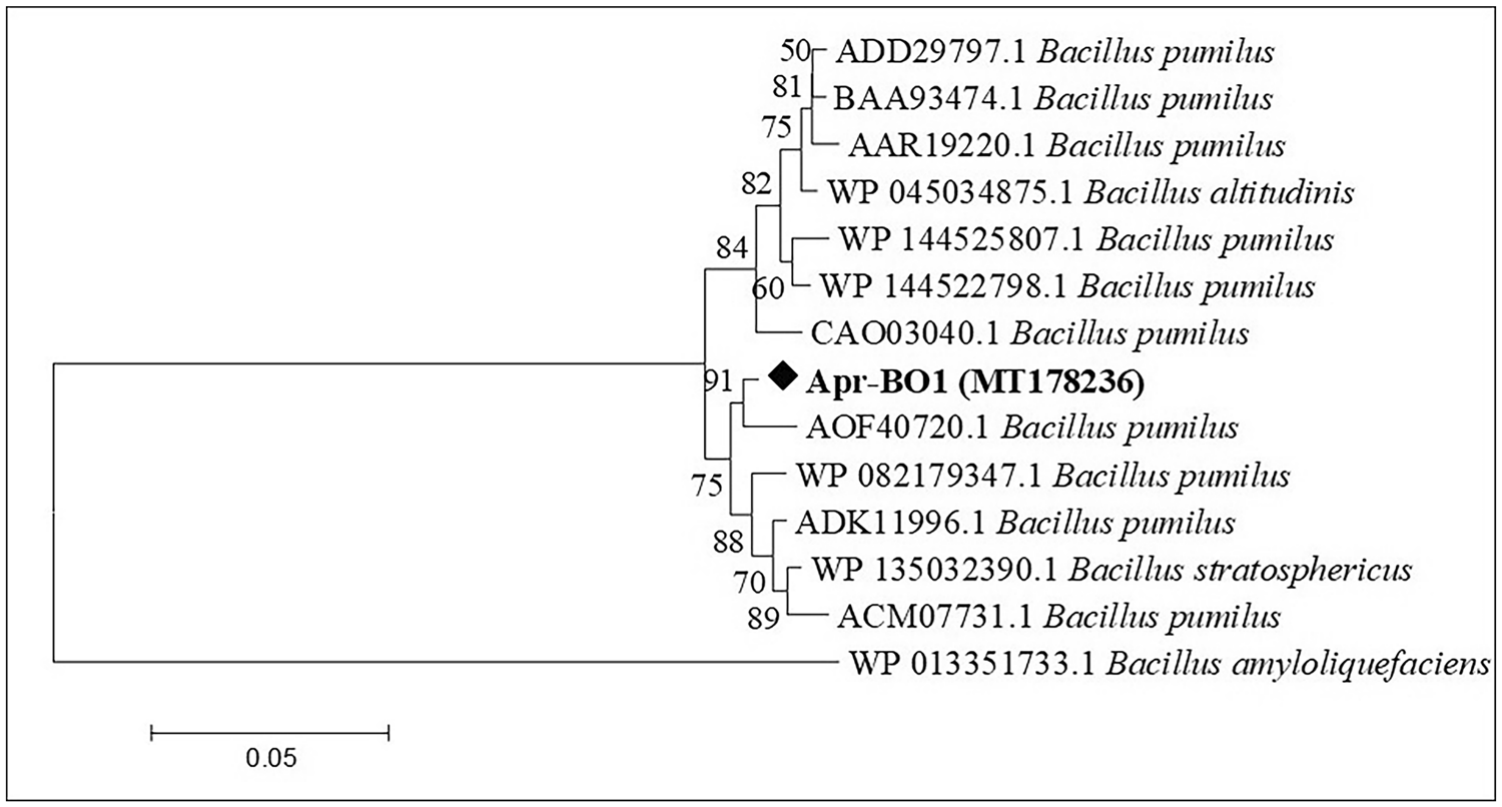
Phylogenetic analysis of cold-active Apr-BO1 with other highly identical alkaline proteases, using amino acid sequences. MEGA 7.0 software was used to deduce the evolutionary history. Next to branches are the bootstrap tests with 1000 replicates.

**Figure 4:**
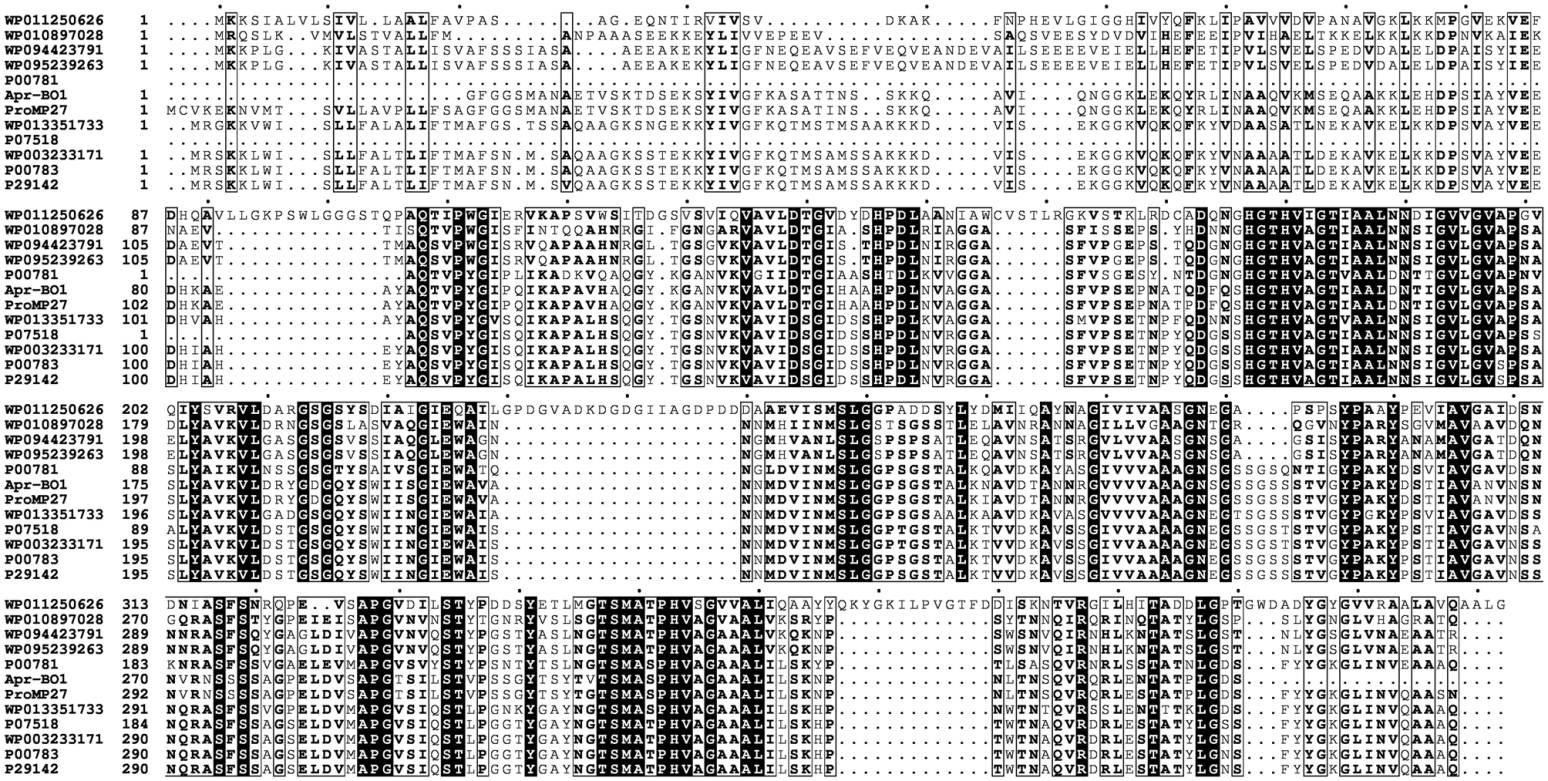
Analysis of amino acid sequences by multiple alignments using homologous proteases. The residues highlighted by the black background are strictly conserved, and those boxed are conservatively substituted. The figure was produced using ESPript 3.0 program^88^.

Comparing it with the NCBI database, the nucleotide sequence of amplified protease gene (Supplementary Fig. S3) showed high similarity (96%) with serine alkaline proteases of *Bacillus pumilus*. The protease gene was provided GenBank accession no. MT178236. The inferred protein sequence of Apr-BO1 showed high similarity to serine peptidase S8A subfamily from *Bacillus* species (Fig. 3). As per the MEROPS database (http://merops.sanger.ac.uk/) phylogenetic classification and sequence analysis, it was concluded that Apr-BO1 belongs to S8A subfamily of serine alkaline protease. Protein sequence alignment in Fig. 4 contains sequence alignment of Apr-BO1 and other homologous subtilisins (S8A subfamily) like, Pro-TK-SP from *Thermococcus kodakarensis* (GenBank No. WP011250626), alkaline protease from *Alkalihalobacillus halodurans* (GenBank No. WP010897028), alkaline protease from *Alkalihalobacillus clausii* (GenBank No. WP094423791), alkaline protease from *Bacillaceae* (GenBank No. WP095239263), subtilisin Carlsberg from *Bacillus licheniformis* (GenBank No. P00781), ProMP27 from *Bacillus pumilus* (GenBank No. KX431582), serine protease from *Bacillus subtilis* (GenBank No. WP013351733), AprE from *Bacillus pumilus* (GenBank No. P07518), AprE from *Bacillus* (GenBank No. WP003233171), subtilisin amylosacchariticus from *Bacillus subtilis subsp. amylosacchariticus* (GenBank No. P00783) and subtilisin J from *Geobacillus stearothermophilus* (GenBank No. P29142). Besides, it shows the conserved and conservatively substituted regions of the Apr-BO1 protein sequence. The Apr-BO1 consisted of 346 amino acids with theoretical isoelectric point (pI) of 6.5.

### Protein modelling

**Figure 5:**
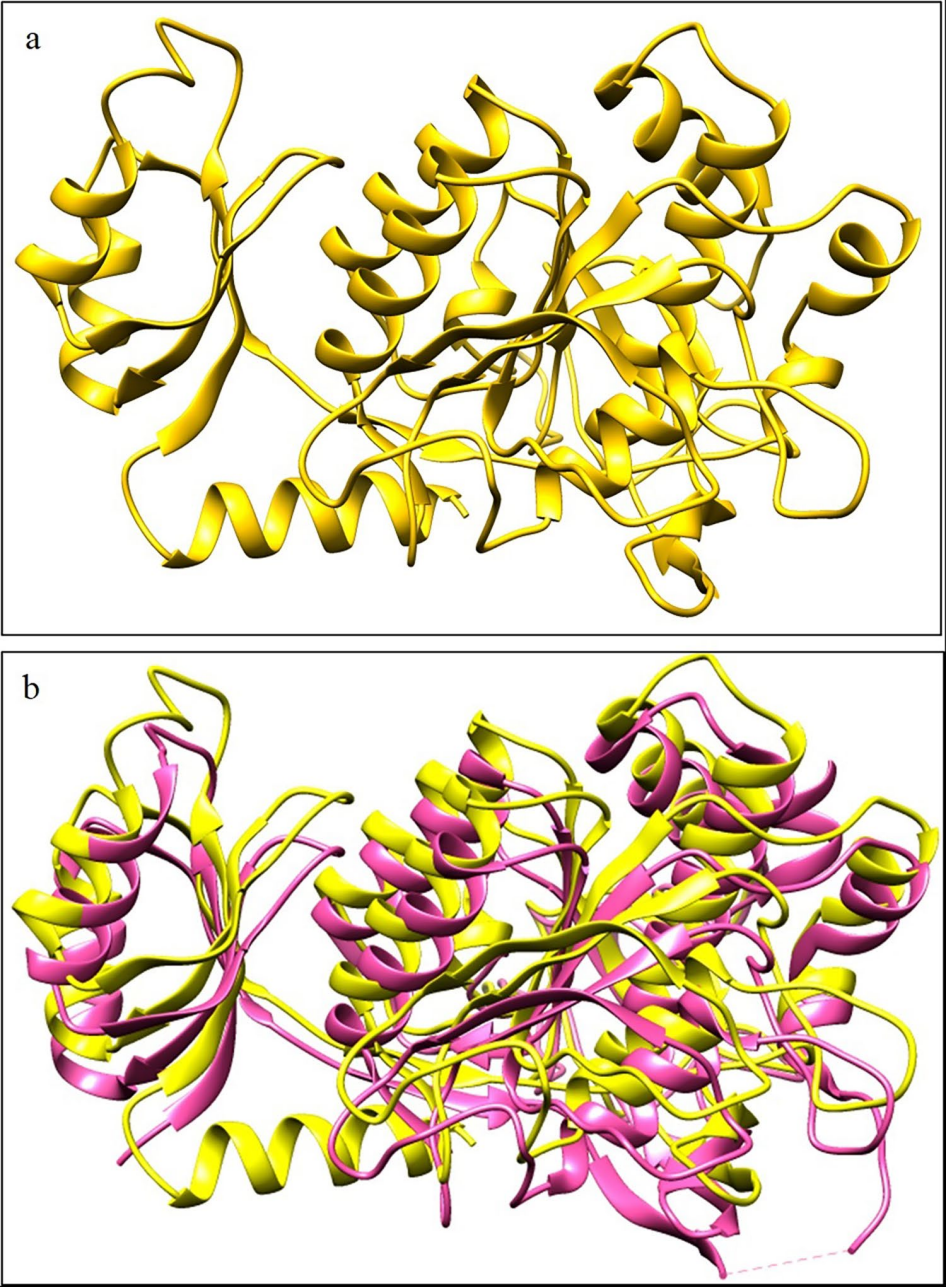
(**a**) UCSF Chimera rendered a refined model of alkaline cold-active protease (Apr-BO1). Model generation was done by GalaxyTBM and among the five predicted models the best one was refined by GalaxyRefine. (**b**) Superimposed structures of Apr-BO1 model (yellow colour) and template (PDB ID: 3WHI) in hot pink. This ID corresponds to subtilisin E of *Bacillus subtilis*. Root mean square deviation (RMSD) was calculated between template and model using the Chimera tool, and it was observed that RMSD between 323 pruned atom pairs is 0.372 Å. RMSD is a quantitative estimate of similarity between two protein structures. Lower the value of model-template RMSD better is the model quality.

**Figure 6:**
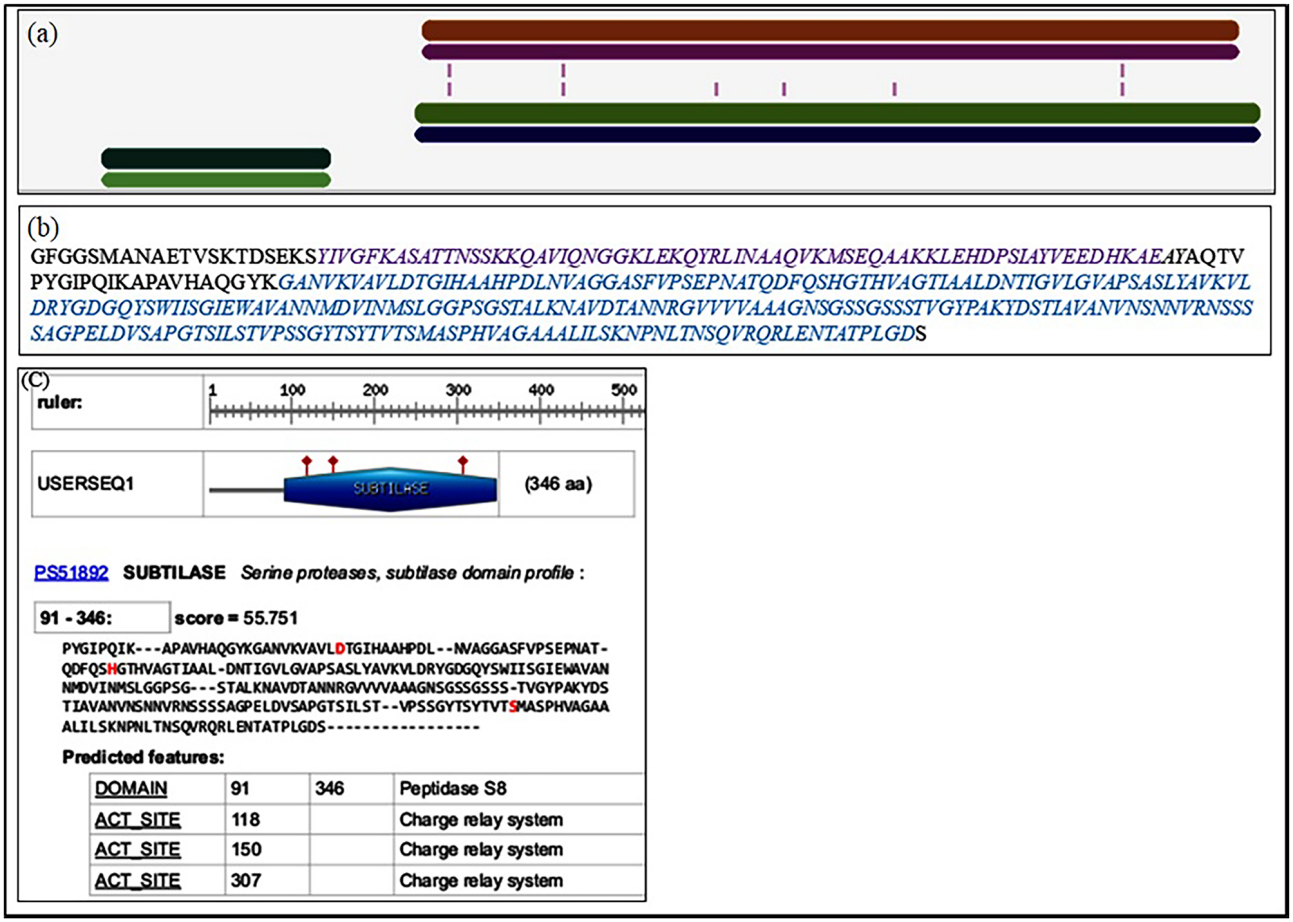
Analysis of domains and active site region of Apr-BO1. This protease showed two domains after InterPro Scan. (**a**) These domains are inhibitor 19 (ranging from amino acid residue 21–84) shown in purple and peptidase S8 domain (from 109 positions to 345th residue) represented in blue. (**b**) The subtilase ranges from 91 to 346 amino acids, and it is known that the S8 family possess a catalytic triad composed of Aspartate/Histidine/Serine, unlike the S53 family, which have glutamate instead of histidine as a part of this triad. ScanProsite tool revealed aspartate 118, histidine 150 and serine 307 as the catalytic triad residues. (**c**) This triad functions in a charge relay mechanism crucial for transforming substrate to products.

The refined model was developed from template subtilisin E of *Bacillus subtilis* (PDB ID: 3WHI). This model (Fig. 5a) qualified the stereochemical quality check, as shown by Ramachandran plot of the refined model where 90.6% of the residues occurred in most favored regions, and 8.1% of the residues were found in additional allowed regions (Supplementary Fig. S4). Further, the model passed Verify 3D analysis with high success as indicated by the favourable averaged 3D-1D score (Supplementary Fig. S5a). Moreover, the results of ProSA-web were highly encouraging, and the z-score of this model (− 8.81) was found to be well within the reference range, indicating the absence of errors (Supplementary Fig. S5b). Additionally, the root means square deviation value of model and template (3WHI) was 0.372 Å again stamping model quality (Fig. 5b). Our InterPro investigation revealed two domains of Apr-BO1, and further analysis by ScanProsite showed subtilase region (91–346). ScanProsite tool revealed aspartate 118, histidine 150 and serine 307 as the catalytic triad residues (Fig. 6).

### Industrial application

#### Wash analysis and detergent stability

**Figure 7:**
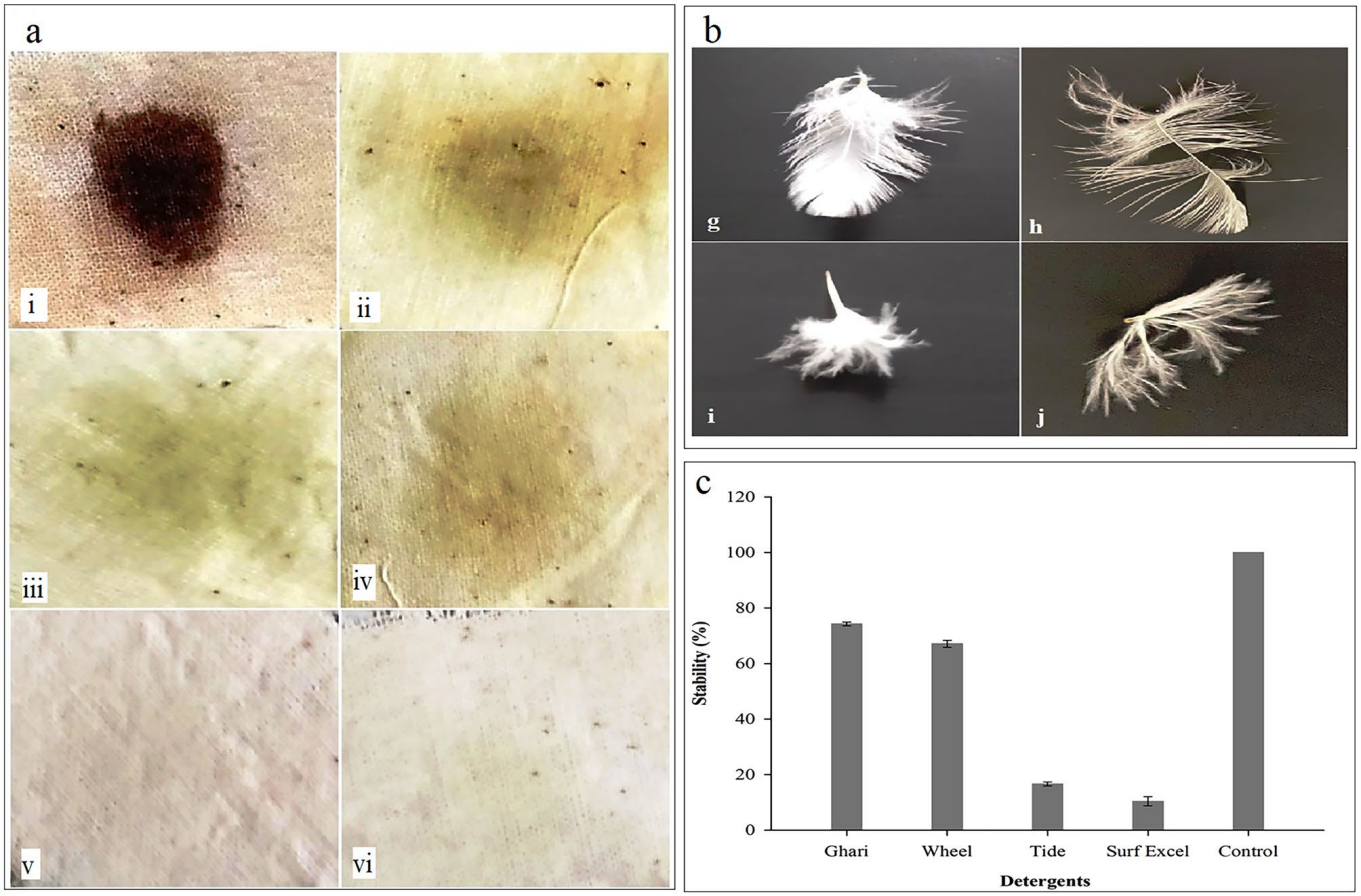
(**a**) Application of purified Apr-BO1 and commercial detergents (Tide and surf Excel) on blood-stained cloth. The treatments given include: (i) untreated cloth, control; (ii) Apr-BO1 only; (iii) Detergent (Tide only); (iv) Detergent (Surf Excel only); (v) Detergent (Tide) + Apr-BO1; (vi) Detergent (Surf Excel) + Apr-BO1. (b) Visual observation of feather fibril detachment when Apr-BO1 enzyme treatment was performed for 8 h at 20 °C. The dehaired feather is shown in the right (h,j), whereas control (without treatment) is shown in the left (g,i). (**c**) Detergent stability of purified Apr-BO1.

Bloodstain was removed in the combination of Apr-BO1 and commercially available detergent (Tide and Surf excel). Comparing the stain removal efficiency of Apr-BO1 with detergents showed better results compared to Surf excel and almost equal stain removal efficiency compared to Tide (Fig. 7a). While as detergent stability test revealed good stability with two out of four commercial detergents (Fig. 7c). Stability of 74% and 67% was observed with Ghari and Wheel, respectively, while only 16.63% stability was observed with Tide and 10.0% with Surf excel.

#### Feather hydrolysis

The detachment of fibril from the feather branch (barb) was visibly observed (Fig. 7b) after the chicken feather was incubated for 8 h in the enzyme (100 µg/ml) at 20 °C. The results showed that it could prove effective in industrial dehairing processes at low temperatures.

## Discussion

Given that very little is known regarding the microbial communities of Himalayan glacial soils, the present study reporting the potential proteolytic psychrotrophic microbial strain from Thajwas glacier is presumably the first report from northwestern Kashmir Himalaya. We were able to isolate a psychrotrophic *Bacillus pumilus* BO1 from these soils capable of producing Apr-BO1, a cold-active serine alkaline protease. Previously, few cold-active enzymes have been characterized from psychrophilic and psychrotrophic *Bacillus* species for their industrial uses^4,24^. However, there is still a demand for a cold-active enzyme that can show tolerance to low temperature, alkaline pH, surfactants, oxidizing agents and organic solvents. As per the literature, ours is the first cold-active serine protease with such properties. Apr-BO1 retained its stability and activity under alkaline conditions over a broad array of pH (7.0–11.0), exhibiting its optimal pH at 9.0. This enzyme also retained its stability and activity at low temperatures (5–20 °C) in the presence of several organic solvents. Further, it retained sufficient activity in the presence of several surfactants, oxidizing agents and metal ions than earlier reported cold-active alkaline proteases (CAAP) from *Bacillus*^25,26^, *Stenotrophomonas*^27,28^ and *Pseudoalteromonas*^29^.

Apart from these properties, a high yield of the enzyme at low temperature is deemed highly significant for industrial applications^30^. The CAAP isolated from psychrophilic and psychrotrophic *Bacillus* species has either low yield per hour of cultivation time^25,26^ or are active at temperatures above 20 °C^14^. Apr-BO1, on the other hand, was found to have a high yield at a low temperature (20 °C), making it the first CAAP from psychrotrophic *Bacillus pumilus* BO1. The purification results are also higher (Table 1) than what has been reported earlier for CAAP isolated from *Bacillus*^25,26^. The molecular weight (38 kDa) of Apr-BO1 is comparable to what has been reported so far, such as serine alkaline proteases isolated from *Bacillus pumilus* CBS^31^, *Bacillus* sp. RKY3^32^ and *Bacillus subtilis* WLCP1^25^.

The tolerance to organic solvents is crucial for the enzyme to withstand harsh industrial processes^8^. In agreement with Shah et al.^33^ mentioning that CAAP isolated from *Bacillus cereus* AK1871 retained maximum activity with organic solvents like benzene, toluene and xylene, Apr-BO1 for the present study also retained its maximum activity in their presence (Table 4). This tolerance suggests its potential application in peptide synthesis. Besides, the Apr-BO1 retained 85.0 and 71.0% (Table 3) activity on treatment for 30 min with 0.5 and 1.0% SDS, respectively. Previous studies on the stability of S8 subtilisins with SDS showed almost similar observations in *Bacillus* sp. JB-99 protease^34^, alkaline proteases from *Bacillus clausii* I-52^35^, *Bacillus subtilis* RD7^36^, and novel *Bacillus caseinilyticus*^37^. Inhibition observed in Apr-BO1 enzyme for the present study was not as sturdy as reported for various serine alkaline proteases from *Bacillus pumilus* AI^38^, *Bacillus* sp. ZJ1502^39^, *Bacillus safensis* S406^40^ and *Bacillus invictae* AHI^41^, adds to its novelty.

Serine proteases are generally inactivated by H_2_O_2_ due to the oxidization of methionine^18,42,43^.

However, Apr-BO1 remained stable in the presence of H_2_O_2_, and its activity was marginally increased by 3.5% and 5.8%, respectively, at 0.5% and 1.0% (v/v) H_2_O_2_ (Table 3). Thus it appears comparable with other novel serine alkaline proteases like SBcas3.3 from *E. coli*^44^, Aprx-SK37 from *Virgibacillus* sp. SK37^43^, Apr-PCC from *Idiomarina* sp. C9-1^42^ and oxidase stable protease from *Bacillus cereus* SV1^45^. It retained its activity for 30 min that presents its unique nature to withstand H_2_O_2_ incubation for a longer duration than others. Contrary to earlier reports that Hg^2+^ and Cd^2+^ exert an inhibitory effect on proteases as Hg^2+^ reacts with thiol group residues, histidine and tryptophan^42,46^, the results of the present study showed that Hg^2+^ and Cd^2+^ had an impact on enzyme activity but did not completely inhibit it (Table 2). For other metal ions like Fe^2+^ and Cu^2+^, no influence on the enzyme activity was recorded. These ions slightly increased the enzyme activity at lower concentrations (Table 2). Similarly, Apr-BO1 showed increased activity with increasing Mn^2+^ concentration, which has also been observed in alkaline serine proteases from *Bacillus pumilus* TMS55^47^, *Bacillus acquimaris* VITP4^48^, *Bacillus circulans* L.^49^, *Bacillus* strain SH1^50^ and proteases from *Bacillus laterosporusn*^51^. Ca^2+^ also showed the stimulating effect on the enzyme activity at 1.0 mM to 10.0 mM concentration. Alkaline protease from *Virgibacillus pantothenticus*^52^, a serine protease from novel *Bacillus lehensis*^53^ and alkaline protease from *Bacillus altitudinis* W3^54^ also reported similar stimulating effects. S8 proteases have previously been activated by Ca^2+^ and inhibited by EDTA. According to previous research on subtilisin, Ca^2+^ plays an important structural role in the enzyme, and the effect of Ca^2+^ on enzyme activity may be due to the formation of a stable active conformation^54^. Subtilisin has two binding sites, one strong and one weak, in most cases^21^. As a result, the Apr-BO1 structure may have Ca^2+^ binding sites.

Moreover, in comparison to previous reports suggesting proteases from *Bacilli* had low K_m_ between 0.57 and 4.0 mg/ml at higher temperatures (45–60 °C)^41,55–57^, the Apr-BO1 had a low K_m_ (1.0 mg/ml) and V_max_ (10.0 µmol/ml/min) at low temperature (20°), which indicates that it has high substrate affinity and efficient catalytic activity. The sequence of Apr-BO1 and other similar proteases showed low similarity after comparing with the database available at UniProt (https://www.uniprot.org/blast/). The highest identity of 96.0% was observed with *Bacillus pumilus* SARF-032, displaying the novel protease nature of Apr-BO1. The Ramachandran Plot of the refined model showed that 90.6% residues occurred in most favored regions while as 8.1% of the residues occurred in additional allowed regions (Supplementary Fig. S4). This is significant, and good quality model possesses above 90% residues in the most favored regions^58,59^. The quality check through ProSA further confirmed the model accuracy as its z-score was well within the confines of z-scores of experimental structures possessing similar size^60,61^. Our study showed that Asp 118/His 150/Ser 307 constitutes this protease’s catalytic triad. These results parallel with the findings where it has been demonstrated that S8 proteases have Asp/His/Ser as a catalytic triad, unlike S53, which have glutamate instead of histidine (Fig. 6). Serine proteases use a charge relay mechanism for catalysis, and these catalytic triad residues play a key role in this mechanism^62^. Besides, the predicted pI of the 11 subtilisin-like serine proteases and their molecular mass in comparison to the predicted pI (6.5) and molecular mass of Apr-BO1 showed that the molecular mass of Apr-BO1 was almost the same as that of other proteases except few. At the same time, two proteases (WP010897028 and P07518) had pI similar to Apr-BO1 (see Supplementary Table S1). Previous studies show that pI of most of the S8 proteases varies between 4 and 9^54,63^.

Apr-BO1 efficiently removed bloodstain from the cloth without the assistance of any detergent (Fig. 7a). This compares well with previous results showing similar observations in mesophilic *Bacillus pumilus*^8^ and psychrophilic *Bacillus subtilis*^25^. The stain removal efficiency of Apr-BO1 to that of previously reported alkaline proteases^28,64–66^ shows that Apr-BO1 has higher efficiency at low concentrations (100 µg/ml) and low temperatures (20 °C), which makes it an appropriate detergent additive. Further, it also demonstrated good detergent stability, which reveals that it can be used in detergents and other industrial processes to carry out operations at lower energy expenditures. The Apr-BO1 also showed promising results in detachment of chicken feather after 8 h incubation at 20 °C in 4U of the enzyme. Similar results were obtained after 8 h incubation of chicken feather in 4U of enzyme along with 0.1% beta-mercaptoethanol (βME) at 45 °C and after 24 h incubation without βME. When compared with the previously isolated keratinolytic serine protease from thermophilic *Bacillus subtilis*^67^. The present study is the first report of such a kind from psychrotrophic bacteria. This enzyme activity could provide an energy-saving alternative to enzymes from thermophilic bacteria where the temperature of the substrate is raised so that the enzyme could function effectively^4^.

Novel, environmentally friendly enzymes are in high demand for industrial processes to reduce rising industrial costs and pollution. CAAP are emerging as essential enzymes with high commercial value in the dehairing and detergent industries due to their high substrate affinity at lower temperatures. The results from present study show that Apr-BO1 exhibits a high yielding capacity over a short duration (48 h), with a low K_m_ value. It indicates that it exhibits a high affinity towards the substrate at low temperature, highlighting its novel behaviour. In addition, Apr-BO1 was active and stable over a wide pH and temperature range and retained its maximum activity with various metal ions, surfactant and organic solvents. This unparallel catalytic nature makes it suitable for industrial purposes. The modelled and certified structure of this protease may provide opportunities for further studies on this enzyme.

## Material and methods

### Sample collection and culture conditions

Soil samples were collected from Thajwas Glacier (34° 21′ 53.7′′ N latitude and 75° 21′ 03.6′′ E longitude) Kashmir, India at an altitude of 3900 m (a.m.s.l) in sterile poly-bags, plastic vials and transported to the laboratory^68^. A sixfold dilution of the soil sample was prepared, and the isolation of bacterial strain was done according to Zhang et al.^69^. Further, the initial proteolytic screening of the isolated bacterial strains was carried out on skim milk agar^70^.

### Identification of psychrotrophic bacterial strain BO1

The cell morphology of BO1 strain was studied by Gram’s stain under a light microscope (Olympus IX71, Japan). The genomic DNA was extracted using the bacterial genomic DNA isolation kit GenElute (Sigma-Aldrich, USA), and for molecular identification, 16S rRNA gene amplification was done by PCR^69^. The 16S rRNA gene sequence was matched with the NCBI database using BLASTn program. The alignment of nucleotide sequences of BO1 strain and that of similar species was done by ClustalW. The phylogenetic tree for the 16S rRNA gene was constructed by the Neighbor-Joining method in MEGA 7.0^71^.

### Protease production

#### Qualitative screening

Qualitative screening of BO1 strain for protease production was done on three different media (agar 1.5% w/v, peptone 0.5%, NaCl 0.5% w/v, beef extract 0.3%) containing skim milk, casein and gelatin (1% w/v) as protein source separately. The media plates were incubated at 20 °C for 48 h, followed by submerging the bacterial colonies in mercuric chloride reagent and observing the zone of hydrolysis^65^.

#### Quantitative screening

The culture media contained: peptone (0.75% w/v), casein (1% w/v), MgSO_4_·7H_2_O (0.5% w/v), KH_2_PO_4_ (0.5% w/v), glucose (0.5% w/v) and FeSO_4_·7H_2_O (0.01% w/v)^65^ and pH was adjusted at 9.0 using sterilized Tris–HCl buffer. The overnight grown bacterial culture was inoculated in the ratio of 5:100 into protease production media, and media flasks were incubated for 48 h in a shaking incubator at 20 °C, 150 rpm. The media was centrifuged at 16,128×*g* for 10 min at 4 °C to collect the supernatant containing crude extracellular enzyme, and the purification of the enzyme was carried out.

#### Purification and characterization of alkaline protease

The protein aggregate and non-protein polymers were separated from the supernatant by adding ammonium sulphate to 40% saturation. Ammonium salt was further added up to 80% saturation to recollected supernatant. Centrifugation for both saturations was carried out for 10 min at 16,128×*g* and 4 °C^72^. 50 mM Tris–HCl buffer (pH 9.0) was utilized to dissolve the protein precipitate, and dialysis was carried out to remove ammonium sulphate residue from protein precipitate using a dialysis bag (Sigma Aldrich, USA). The resultant dialysate was loaded onto the Sephadex G-50 superfine column (Sigma-Aldrich, USA). Collected pooled fractions were saturated up to 60% with pre-chilled acetone (added slowly with gentle mixing) and kept at 4 °C for 4 h to precipitate^73^.

#### Electrophoresis

The SDS-PAGE^74^ was used to determine the molecular mass of the purified Apr-BO1, with 5% stacking gel and 12% resolving gel. The protein bands were stained by Coomassie Brilliant Blue R-250 and a standard protein marker (Thermo scientific PageRuler) was used as a reference.

#### Protein determination and protease assay

Protein content (mg/ml) was determined following Lowry et al.^75^ using bovine serum albumin (BSA) as a reference after each stage of the enzyme purification process. While as the proteolytic activity was measured by a modification of Zhou et al.^42^. The reaction mixture containing 0.6% (w/v) casein was dissolved in 200 µl 0.05 M Tris–HCl buffer (pH 9.0) and 100 µl enzyme. The reaction mixture was incubated at 20 °C for 30 min, and the reaction was stopped by adding 300 µl of 10% (w/v) trichloroacetic acid (TCA) while for control (blank) TCA was added just before incubation. Centrifugation of the reaction mixture was done for 10 min at 16,128×*g* and 4 °C, and 500 µl of supernatant was pipetted out into a new tube to which 2 ml of Na_2_CO_3_ (0.4 M) and 250 µl of Folin–Ciocalteu reagent (1 N) were added. Incubation of the mixture was done for 25 min at 30 °C and absorbance was measured at 660 nm. Protease activity (1U) was defined as the amount of enzyme (Apr-BO1) needed to release 1.0 µmol of tyrosine for every ml for each minute under standard assay conditions. The amount of tyrosine released was determined by the tyrosine standard curve.

### Effect of pH and temperature on the protease activity and stability

The optimal pH of Apr-BO1 was assayed at 20 °C in 0.1 M Phosphate buffer (pH 6.0–7.0), 0.1 M Tris–HCl (pH 8.0–9.0) and 0.1 M Glycine–NaOH (pH 10.0–12.0) containing 0.6% casein (w/v). The effect of pH on enzyme stability was assayed by pre-incubating enzyme in the said buffers for 1 h at 20 °C. The % residual activities (i.e., sample activity after incubation/activity of sample before incubation × 100) were then measured.

The temperature was assayed between 5 and 40 °C with a gradient of 5 °C for 30 min in 0.1 M Tris–HCl buffer (pH 9.0) containing 0.6% casein (w/v). The temperature stability was determined by assessing enzyme residual activity by pre-incubating the enzyme at different temperatures (5–40 °C) with the gradient of 5 °C for 1 h in 0.1 M Tris–HCl buffer, pH 9.0.

### Effect of metal ions and other reagents on the protease activity

The effect of various metal ions (Hg^2+^, Co^2+^, Fe^2+^, Cd^2+^, Cu^2+^, Mg^2+^, Mn^2+^, Ca^2+^ and Zn^2+^) on the enzyme catalytic activity was studied by assaying the relative activity with the addition of 1.0 mM, 5.0 mM and 10.0 mM concentration of metal ions. Similarly, the effect of ethylenediaminetetraacetic acid (EDTA) on enzyme activity was analyzed by the addition of 1.0 mM, 5.0 mM and 10.0 mM concentration. In addition, the effect of organic solvents (benzene, toluene and xylene), surfactants (Triton-x-100, SDS and Tween-80) and oxidizing agent (H_2_O_2_) on relative activity of enzyme was analyzed by adding, 0.5% and 1.0% (v/v; w/v for SDS) concentrations of organic solvents, surfactants and oxidizing agent to 500 µl of Tris–HCl buffer (pH 9.0) containing 0.6% casein (w/v). The stability of enzyme against organic solvents (benzene, toluene and xylene) was assessed by pre-incubating 100 µl of the enzyme at 20 °C in each of these solvents for 30 min and assayed accordingly.

### Substrate efficacy

In order to check the substrate efficacy of enzyme, a concentration of 1.0% of four substrates (casein, gelatin, BSA and skim milk) was assayed at 20 °C and pH 9.0.

### Enzyme kinetics of cold-active Apr-BO1

Enzyme kinetic parameters of Apr-BO1 were calculated at 0.02–0.22 g% concentrations of casein at pH 9.0 and temperature 20 °C for 30 min. Lineweaver- Burk plot^76^ was used to calculate the K_m_ and V_max_ of the cold-active protease against casein.

### Amplification of alkaline protease gene

The conserved protein gene (apr) encoding the Apr-BO1 was amplified using a pair of primers (AprF: ATGTGCGTGAAAAAGAAAAATGTG and AprR: TTAGTTAGAAGCTGCTTGAACGTT) designed at NCBI’s Primer Blast tool. Genomic DNA already extracted for the identification of BO1 strain was now used again as a template for the amplification of alkaline protease gene by PCR with similar PCR conditions used for the 16S rRNA gene. The translated amino acid sequence of the Apr-BO1 gene was analyzed in the NCBI BLASTp program, and the sequences were aligned using Clustal Omega. Phylogenetic tree for protein sequence was constructed using MEGA 7.0 with Neighbor-Joining method^71^. The theoretical isoelectric point (pI) of Apr-BO1 was calculated at https://web.expasy.org/cgi-bin/compute_pi/pi_tool.

### Protein modelling and refinement

GalaxyTBM was used for producing the protein structure from a given amino acid sequence. It first generates the structure of reliable core region through a template-based approach, after which the unreliable local regions are recognized and re-modelled through ab initio method^77,78^. Among five predicted models, the best one was chosen for refinement. GalaxyRefine—another service of GalaxyWEB was used to improve the quality of model generated^79^. The refined model was rendered with the help of UCSF Chimera (1.10.2) software^80^. For generating this model, the template used was subtilisin E of *Bacillus subtilis* (PDB ID: 3WHI)^81^. Root mean square deviation (RMSD) was estimated by superimposing the model on a template using MatchMaker of Chimera software. Other valuable information regarding this protease, including several domains and catalytic triad, was investigated using the pandemically popular tools InterPro and ScanProsite^82,83^.

### Model validation through different approaches

The stereochemical quality of the model was checked by PROCHECK, and the model compatibility with its sequence was evaluated by Verify 3D^59,84,85^. The errors in the refined model were examined using Protein Structure Analysis (ProSA)-web^86^.

### Industrial application

#### Detergent stability and wash analysis

The stability of cold-active enzyme with four commercially available detergents viz; Surf Excel, Wheel (Hindustan Unilever Limited), Tide (Procter & Gamble) and Ghari (RSPL Limited) was assayed by incubating 100 µl of enzyme in 200 µl of 7.0 mg/ml detergents solution. The detergent solution was incubated at 100 °C for 1 h to deactivate the already present proteases in the detergents before adding the enzyme.

To check the stain removal efficiency of the enzyme Furhan et al.^25^ was followed with modification. White cotton cloths measuring 4 cm × 4 cm were stained with blood and dried. The stained pieces of cloths were given following treatments: (a) 400 µl of purified enzyme (100 µg/ml) + 20 ml of tap water + blood-stained cloth; (b) 400 µl of purified enzyme + 20 ml of detergent solution (0.07% w/v) + blood-stained cloth; (c) 0.07% (w/v) detergent solution + blood-stained cloth and (d) bloodstained cloth + 20 ml of tap water was used for blank control. The treatments were given in triplicates and incubated at 20 °C for 30 min. The cloth was then rinsed with tap water, dried and evaluated for stain removal efficiency.

#### Feather fibril detachment

Hydrolysis and detachment of feather fibril by Apr-BO1 was carried^87^ with few modifications. The chicken feathers were acquired from a local slaughterhouse and washed first with tap water, rinsed with distilled water and sterilized by autoclaving. The feathers were left overnight to dry at room temperature and incubated in 2 ml of the purified enzyme with 100 µg/ml concentrations for 8 h at 20 °C, washed and dried.

### Ethical statement

The authors in the current study carried out no animal or human studies. The authors further confirm that this work is original and has not been published, nor is it currently under consideration for publication elsewhere.

## Supplementary Information

Supplementary Information.

## Data Availability

Data about the 16S rRNA gene sequence and Apr-BO1 gene sequence have been deposited at NCBI GenBank under the accession number MN094861 and MT178236, respectively.
