# Supplementary material for: Isolation and characterization of a new cold-active protease from psychrotrophic bacteria of Western Himalayan glacial soil
Source: Sci Rep. 2021 Jun 17;11:12768. doi: 10.1038/s41598-021-92197-w (PMC8211794; doi:10.1038/s41598-021-92197-w)
Supplement: Supplementary file 1 — Supplementary Information. [file 41598_2021_92197_MOESM1_ESM.docx]

**Scientific Reports**

**Isolation and Characterization of a New Cold-Active Protease from Psychrotrophic Bacteria of Western Himalayan Glacial Soil**

Saleem Farooq^1,2^, Ruqeya Nazir^2^*, Shabir Ahmad Ganai^3^ and Bashir Ahmad Ganai^2^**

^1^Department of Environmental Science, University of Kashmir, Jammu and Kashmir, India.

^2^Centre of Research for Development (CORD), University of Kashmir, Jammu and Kashmir, India.

^3^Division of Basic Sciences and Humanities, FoA, SKUAST-Kashmir - 193201, Jammu & Kashmir, India.

**Corresponding Authors**

*Dr. Ruqeya Nazir

Assistant Professor

Microbiology laboratory, Centre of Research for Development (CORD), University of Kashmir, Hazratbal, Srinagar, Jammu and Kashmir, India.

Email: ruqeya.ku@gmail.com

Phone: +919419023191

**Professor Bashir Ahmad Ganai

Professor

Biochemistry laboratory, Centre of Research for Development (CORD), University of Kashmir, Hazratbal, Srinagar, Jammu and Kashmir, India.

E-mail: [bbcganai@gmail.com](mailto:bbcganai@gmail.com)

Cell no: +919797247851


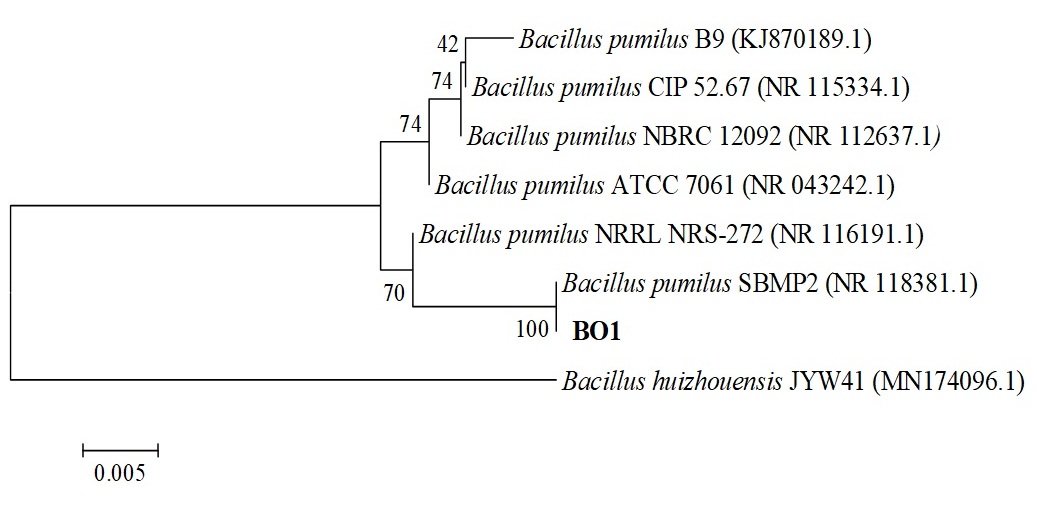


**Figure S1** 16S rRNA phylogenetic tree (Neighbor-Joining) for psychrotrophic BO1 strain. The tree shows the close relationship of BO1 strain with other highly similar *Bacillus pumilus* species and the branches of the tree show the bootstrap test with 1000 replicates.


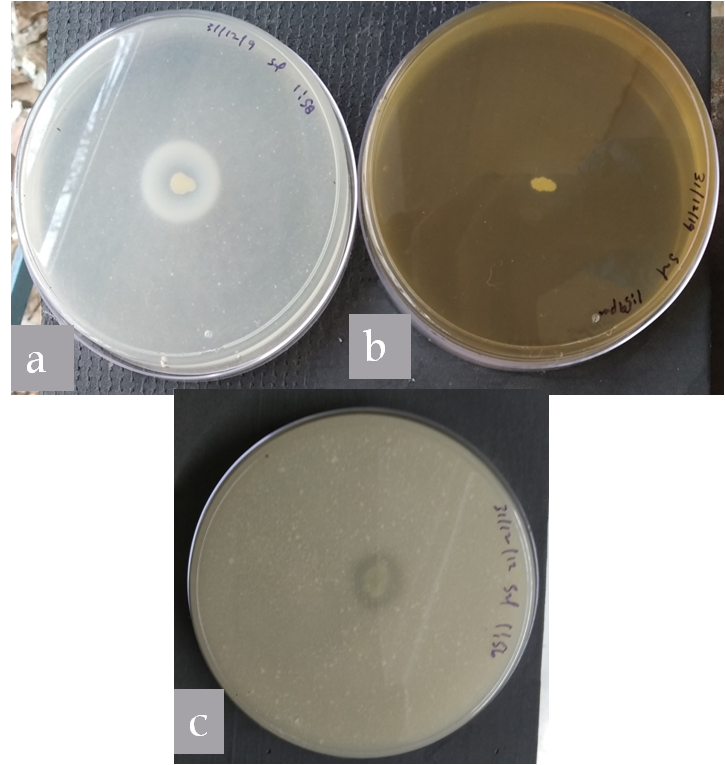


**Figure S2** Zone of hydrolysis on (a) casein (b) gelatin and (c) skim milk.


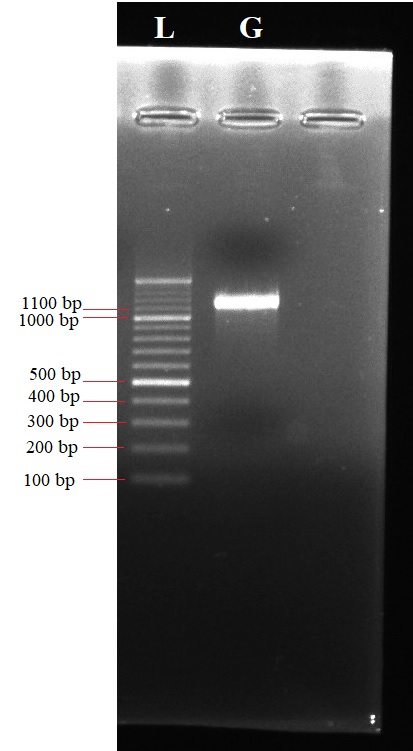


**Figure S3** Amplified protease gene amplicon, lane G and the 100 bp DNA molecular marker, lane L.


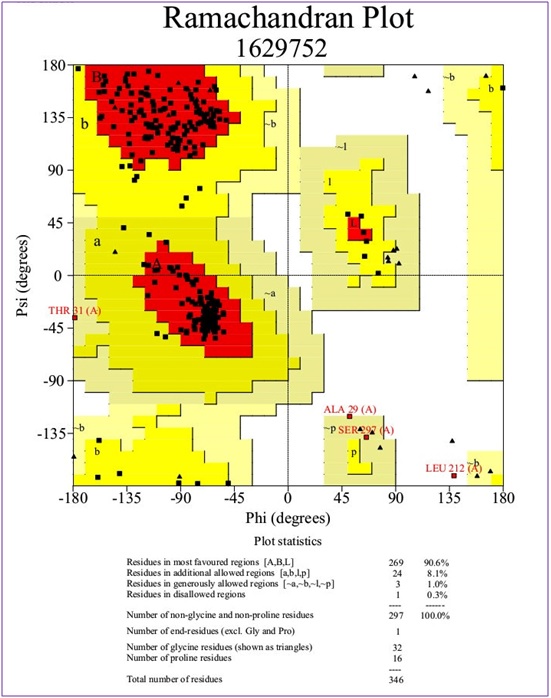


**Figure S4** Ramachandran Plot of refined model of (*Bacillus pumilus* BO1). As the model has 90.6% of the residues in most favoured regions this indicates its good quality. This Plot was generated using the PROCHECK of SAVES v5.0.


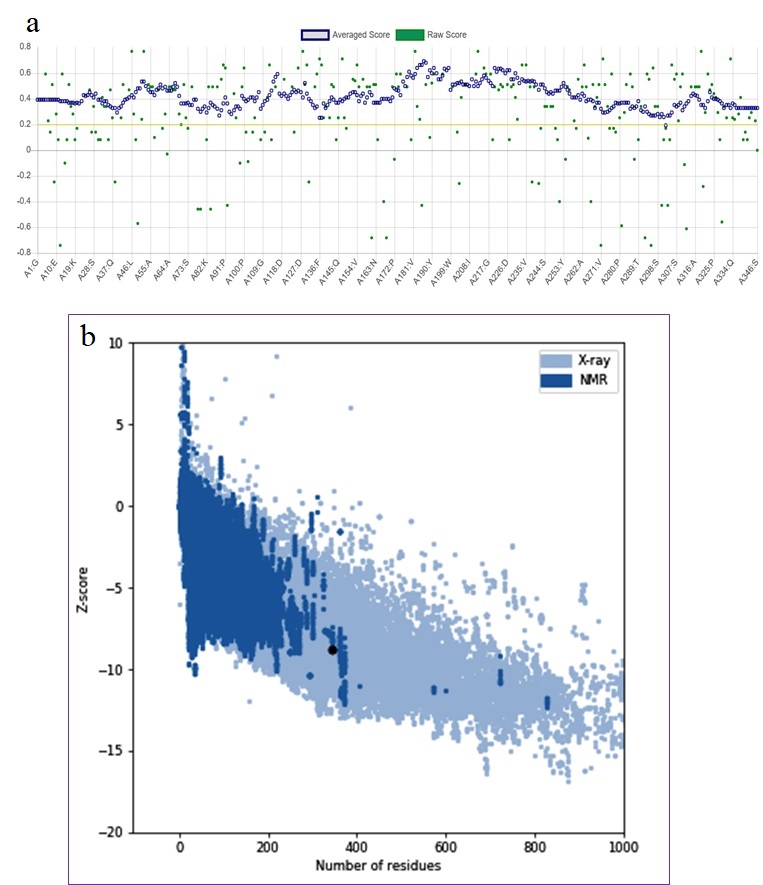


**Figure S5** (a) The 3D-1D compatibility of refined model was checked by Verify 3D. The model successfully passed the Verify 3D check as 99.71% of the amino acid residues showed averaged 3D-1D score >= 0.2. For passing this test minimum 80% of the residues should portray the defined score >= 0.2. (b) The quality of model was also checked by ProSA-web. This easy to use interface of ProSA is used for detecting errors not only in experimentally determined structures but also in theoretical models. From a given input structure, ProSA calculates z-score which in turn indicates the overall quality of the model. The z-score of the model being within the reference range strongly certifies its good quality.


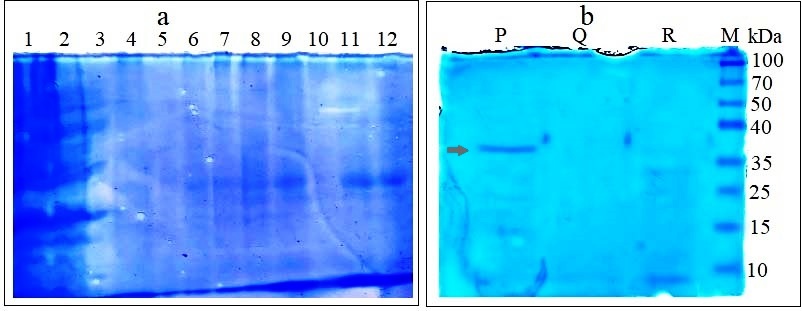


**Figure S6** SDS-PAGE of Apr-BO1. (a) Lane 1 & 2, crude extract; lane 6-9, dialyzed fraction; lane 11 & 12, Sephadex fraction (initial). (b) Lane P, purified Apr-BO1 and lane M, protein marker.

**Table S1** Comparison of predicted isoelectric points (pI) and molecular mass of Apr-BO1 and other subtilisins (shown in figure 4). The molecular mass of Apr-BO1 was almost same as that of other proteases. While as only 2 proteases (WP010897028 and P07518) had pI similar to that of Apr-BO1.

| **GenBank accession numbers** | **Isoelectric point (pI)** | **Molecular mass (kDa)** |
| --- | --- | --- |
| WP011250626 | 4.60 | 43 |
| WP010897028 | 6.56 | 38 |
| WP094423791 | 4.68 | 38 |
| WP095239263 | 4.68 | 38 |
| **MT178236 (Apr-BO1)** | **6.50** | **38** |
| KX431582 | 7.10 | 39 |
| WP013351733 | 9.13 | 39 |
| P07518 | 6.30 | 27 |
| WP003233171 | 9.04 | 39 |
| P00783 | 9.04 | 39 |
| P29142 | 9.04 | 39 |
